# Scaffolded, embedded required: information literacy education in undergraduate health sciences

**DOI:** 10.29173/jchla29666

**Published:** 2023-08-01

**Authors:** Denise A. Smith, Stephanie Sanger

**Affiliations:** 1 Health Sciences Library, McMaster University, Hamilton, ON

## Abstract

**Background:**

The Health Sciences Library and Bachelor of Health Sciences (BHSc) program at McMaster University worked together to build a multi-year information literacy (IL) curriculum embedded within the program under a suite of courses called Praxis Pathways.

**Description:**

Praxis Pathways consists of four Threads. Thread 4: information literacy is the focus of this case report. The authors will describe the multi-year embedded IL curriculum, which is scaffolded to build both IL skills, such as database searching, and introduce students to key conceptual conversations in IL, production, and dissemination.

**Outcomes:**

BHSc program graduates in 2023 will be the first to have completed all four years of the Praxis Pathways courses, including the IL program developed and delivered by the library. The authors will describe how the impact of the program will be evaluated qualitatively and quantitatively going forward.

**Conclusion:**

Embedded librarianship for multi-year, scaffolded IL education in undergraduate programs continues to be a rarity, despite acknowledgement that one-shot instruction has several limitations. The authors present this case report to share how they embedded a for-credit IL curriculum in an undergraduate program that looks beyond the one-shot, skill-based tutorial and focuses on developing adaptive, information literate lifelong learners.

## Introduction

### Background

The Bachelor of Health Sciences (BHSc) program and the Health Sciences Library (HSL) at McMaster University have cultivated a close and collaborative relationship since BHSc launched in 2000. This relationship has fostered a keen awareness of the benefits of information literacy (IL) instruction for undergraduate students and in 2017, the Assistant Dean approached the library with a proposal to include information literacy content in a new four-year course. The Health Sciences Library and the BHSc program worked together to produce a for-credit, scaffolded IL program embedded within a required four-year course curriculum called Praxis Pathways. This course emerged from a desire to introduce a required, multi-year credit to BHSc students that encourages them to engage with the health sciences holistically through a multidisciplinary lens. Curriculum planning began in earnest in the summer of 2018 and Praxis Pathways Level 1 was offered to the approximately 260 incoming BHSc students for the first time in Fall 2019.

In the spring of 2023, 190 students will graduate from the BHSc program. This is the first cohort of students to have completed the full Praxis Pathways curriculum, which includes the first 4-year undergraduate IL curriculum offered within the university’s Faculty of Health Sciences.

This program description details the authors’ objective to develop metaliterate, adaptive lifelong learners and to employ a scaffolded approach to curriculum content development and delivery. Learning outcomes for students, and forthcoming research to measure impact are also discussed.

### Literature Review

IL instruction in undergraduate health sciences education is most popularly discussed in peer-reviewed literature within the context of nursing education. It follows that curriculum-integrated IL and faculty/library collaboration predominantly comes out of nursing education literature [1–6]. In one community college nursing program, the argument for curriculum integration centres around defragmenting IL from nursing instruction, which “requires a paradigm shift from teaching one-time courses…requiring students to build their knowledge and competencies along a novice-to-expert curriculum throughout their educational process” [7]. However, when considering undergraduate education more broadly, there is insufficient high-quality evidence that speaks to the precise benefits and challenges of an integrated and scaffolded IL curriculum when compared to more traditional modes of delivery [8]. Despite some arguments that the impact on students is the same regardless of how IL training is delivered [9], for undergraduate health sciences, the call for a “fully-fledged” curriculum-integrated scaffolded training program is on the rise [10].

This approach to IL education can be of mutual benefit to health sciences librarians and faculty. It creates space for a more holistic approach to IL education that, in addition to teaching applied skills such as database searching, also encourages undergraduate students to engage with higher level theoretical concepts such as information or knowledge equity [11]. There is limited evidence of multi-year IL curricula in undergraduate health sciences education. However, a robust body of literature supports the positive impact of librarian collaboration within courses or assignments on instilling IL within undergraduate students [12–17]. This is complemented by occasional reports of a single course, taught by librarians, that focused on teaching students foundational IL [18,19].

The pursuit of curriculum-integrated IL education has a logical home in undergraduate health sciences, where evidence-based practice is first introduced. In 2016, a proposed curriculum map was published that merges IL, specifically the *ACRL Framework for Information Literacy in Higher Education* [20], and evidence-based practice in an undergraduate health sciences curriculum [21].

## Description

Praxis Pathways is a seven unit longitudinal curriculum that is offered as a full-year course at all levels of the BHSc program. At each level of study, the course content is divided into four threads:

1. Self-knowledge, self-care, and purpose: to explore self-awareness, self-care, and well-being of the self and others
2. Collaboration in community: to explore accountability as a community member and inclusive community building
3. Critical analysis and reflexivity: to explore engaging in critical conversations, and reflexive dialogue
4. Information literacy: to explore information searching skills and understand critical conversations in knowledge creation, dissemination and translation.

Thread 4: information literacy provided the authors, who are academic health sciences librarians, with a unique opportunity to rethink the typical objectives of one-shot IL instruction. Due to their time-limited nature, the one-shot prioritises basic searching skills and provides few opportunities to explore IL conceptually. Using the *ACRL Framework* and adopting the position that IL be reframed as metaliteracy [22], the authors designed a scaffolded four-year program that balances skill-based learning with critical conversations in health information and knowledge dissemination. Each learning module was aligned with key learning objectives and the *ACRL Framework* (see Table 1).

**Table 1:** Scaffolded Information Literacy Curriculum for BHSc Program, McMaster University

Learning Module	Learning Objectives	ACRL Framework
**Year 1**		
Searching for Information I Fall term	Access the library web site and databases Understand what services are available on-site and what services are available online Explore database searching and understand what publication types can be found in a database	Searching as strategic exploration Scholarship is a conversation
Searching for Information II Winter Term	Objectives from Searching from Information I, as well as: Form a research question and identify its main concepts Use main concepts to build a database search in Medline and Web of Science How to use synonyms to broaden a search strategy Construct a database search using Boolean Operators OR and AND	Searching as strategic exploration Scholarship is a conversation
**Year 2**		
Searching for Information III	Objectives from Searching for Information I and II, as well as:	Searching as strategic exploration
Fall Term	Conduct a database search that includes relevant subject headings (MeSH) and key words using the main concepts of a research questions The difference between keywords and subject headings (MesH) Export database search results in Medline and Web of Science	Scholarship is a conversation
Information Dissemination Winter Term	Objectives from Searching for Information I, II, and III, as well as: Scrutinise a news media article reporting on a new study to find key metadata Trace a study reported in the media to its original publication in a library database using metadata mined from the news media article (PubMed, Medline or Web of Science) Use the key concepts of the original study to identify main concepts, build an appropriate database search and retrieve articles Understand key differences between media reports of new knowledge and how new knowledge is reported in academic or clinical literature	Searching as strategic exploration Scholarship is a conversation Authority is constructed and contextual
**Years 3 and 4**		
Introduction to Evidence-Based Practice and the 6S Pyramid	Understand the basics of evidence-based medicine Apply PICO to a patient scenario and form a relevant research question Understand clinical questions and will be able to identify them by type (e.g., diagnostic, therapy) Identify what type of information resource is appropriate for their question, based on its type Select and search an appropriate database or clinical tool based on their perceived information need.	Information has value Authority is constructed and contextual
Advanced database searching	Understand the difference between a basic database search and a comprehensive or robust database search Search multiple clinical and non-clinical	Searching as strategic exploration Scholarship as a
.	databases using all appropriate subject headings and keywords Mine subject heading scope notes to identify appropriate synonyms for keyword searching Translate a single database search into multiple alternative databases Understand the utility of validated search filters and will be able to correctly and appropriately apply a filter for study type using a validated search filter	conversation
Pillars of Information Literacy & CINAHL workshop	Expertly search CINAHL using the skills learned in Years 1 and 2 Understand high-level concepts of IL Draw connections between the theoretical concepts of IL and their information seeking skills and behaviour	Scholarship as a conversation Information has value Searching as strategic exploration
Knowledge Translation & Wikipedia	Understand knowledge translation for consumer health Understand the role of Wikipedia as a potential tool in public health communication Assess the quality of an individual Wikipedia article Add one high-quality reference to an uncited sentence in a Wikipedia article	Information creation as Process
Open Access and the academic publishing ecosystem	Understand standard academic publishing models (traditional vs. open access) Identify the different types of open access publication models (Diamond, Gold, Green, Hybrid) Understand key differences, benefits and drawbacks of publishing models within the academic publishing system	Information creation as process Information has value
Systematic Review searching: the basics	Apply reporting standards to constructing a partial review protocol, focusing on describing methods Compose research question and frame using PICO	Information creation as process Searching as strategic exploration
	Use advanced database searching practices in multiple clinical and non-clinical databases	Scholarship as conversation Research as Inquiry
Measuring evidence impact: impact factors and altmetrics	Describe the meaning of “research impact” Understand the difference between types of metrics (e.g., altmetrics, bibliometrics) How to retrieve impact metrics for individual researchers	Information has value Authority is constructed and contextual
Dissemination of health information and health decision-making	Understand that “information” can take any form Consider the varied social, political and financial impacts of public health information and misinformation	Information has value Authority is constructed and contextual
Best practices for managing data in your research*	What research data management planning describes Understand the research lifecycle Describe research data protection and storage Understand open data movement	Information has value Information creation as process
Health information: Power, privilege, and personal experience*	Understand challenges with hierarchies of evidence in health and medical knowledge Discuss the role of cognitive authority in health information Understand knowledge or information privilege as a concept	Information has value Authority is constructed and contextual

* denotes learning modules that are offered by library partners

The first two years of the curriculum are structured. Students are assigned specific exercises to be completed within a set time frame. Year one focuses on introducing students to the library as a resource for academic research, developing their knowledge about the differences between database searching and using search engines, and introducing students to rudimentary database searching skills, such as how to appropriately use AND and OR operators.

Year two builds on the rudimentary skills introduced in year one using the same model: specific exercises to be completed within a set time frame. These assignments build on the rudimentary skills introduced in year one and students are introduced to subject headings, how subject headings function differently than keywords in database searching, why they are useful, and how to build a search that employs both subject headings and keywords to optimise search sensitivity. Students are expected to finish year two with a strong understanding of why databases are different from search engines, when to use a database instead of a search engine, and how to optimise specific database features to build a comprehensive search. At this stage, database searching is limited to Ovid Medline and Web of Science. Each assignment in year one and year is graded by a librarian or librarian-trained teaching assistant and is integrated with self-reflection to offer students the opportunity to reflect on their learning with each exercise.

The final two years of the curriculum are graded for completion and assumes that all students have established intermediate database searching skills based on their previous two years of study. In years three and four, students are asked to select one or more learning modules from a menu of options provided by each Thread. Learning modules are managed in Microsoft Teams. They can be delivered online or in-person, synchronously or asynchronously. Students have the final two years of their time in the program to select and complete their modules. Thread 4: information literacy modules focus on developing skills more deeply, such as having students building a partial protocol for a systematic review, and providing opportunities for theoretical or abstract conversations about concepts in health and clinical information, such as exploring their understanding of the value of evidence-based medicine in different cultural or societal contexts. Each learning module for the final two years of the program is self-contained. Although developed for the BHSc program, each module is designed so that it can be accessed by any student in any academic program at the university.

## Outcomes

Thread 4: information literacy is the first mandatory, for-credit, multi-year IL program in the Faculty of Health Sciences at McMaster University and such curricula continue to be a rarity in undergraduate health education. In 2022-2023 the program will have been offered to all students in all levels of the program for the first time and the 2023 graduates (n=190) will be the first cohort to have completed the four-year curriculum. All graduates will have completed the required assignments in year one and year two. Table 2 summarizes student participation in the year three and year four learning modules, noting that about half the modules available by the end of the 2022-23 academic year, were not available in the 2021-22.

**Table 2:** Student Enrolment in Year Three and Year Four Learning Modules

Learning Module	2022-2023 Enrolment
Introduction to Evidence-Based Practice and the 6S Pyramid	96
Advanced Database Searching	33
Pillars of Information Literacy & CINAHL workshop	9
Knowledge Translation & Wikipedia	75
Open Access and The Academic Publishing Ecosystem	40
Systematic Review Searching: The Basics	80
Measuring Evidence Impact: Impact Factors and Altmetrics	44
Dissemination of Health Information and Health Decision-making	59
Best Practices For Managing Data In Your Research	21
Graphic Medicine and the Patient Experience	19
Health Information: Power, Privilege, And Personal Experience	19

The effectiveness of this library’s program is currently under investigation. The authors informally recognized a significant improvement from first year to second year with respect to students’ database searching skills, however formal investigation recently received approval from the Hamilton Integrated Research Ethics Approval Board (HIREB). The authors also rely on student course evaluation forms for feedback on the information literacy curriculum, which can guide decision-making in future iterations of the thread.

In early 2023 the authors invited fourth-year students to participate in a study that investigates the impact of Thread 4: information literacy on their learning, their understanding of IL, their perceived confidence in their IL skills. In the fall of 2023, pending ethics approval, the authors may also initiate a multi-year longitudinal analysis that will evaluate students' search skills and understanding of IL over the course of their four-year enrolment in the BHSc program. This study will allow the authors to quantitatively assess the impact of the curriculum on students’ learning.

While the library has historically held a strong relationship with the BHSc program at McMaster, the development of this program has evolved the relationship into an equal partnership where the library is positioned as the authority in information literacy.

At present, the authors intend to offer the learning modules created for BHSc students to other undergraduate, graduate, and professional programs within the Faculty of Health Sciences. This stands to strengthen the library’s position in FHS as key experts in IL education.

## Discussion

In addition to forthcoming studies, subsequent work for the HSL includes the development of additional learning modules for Years 3 and 4. Future learning modules will include opportunities for students to learn about Indigenous health information and knowledge dissemination, the history and evolution of medical evidence, money in medicine and how funding impacts what we know, consumer health information, publication bias, and responsible digital citizenship. Due to the self-contained nature of the learning modules for the latter two years of the BHSc program, there is potential to expand access to additional educational programs at the university and by extension, expand access to a greater number of students.

While helpful at the point-of-need, librarians continue to face challenges with one-shot library instruction. To graduate information literate students who can evolve into adaptive lifelong learners, there is a role for embedded librarianship in undergraduate curriculae. The development of the IL program presented here was possible because the BHSc program invited the library to the table. Getting a seat at the curriculum planning table is a challenge some academic libraries experience. The authors’ plan for qualitative and quantitative evaluations of the program stands to support and impact library efforts to embed their expertise into course curricula at any university or college program.
